# Evolutionary Computation for Expensive Optimization: A Survey

**DOI:** 10.1007/s11633-022-1317-4

**Published:** 2022-01-21

**Authors:** Jian-Yu Li, Zhi-Hui Zhan, Jun Zhang

**Affiliations:** 1 School of Computer Science and Engineering, South China University of Technology, Guangzhou, 510006 China; 2 Pazhou Laboratory, Guangzhou, 510330 China; 3 Victoria University, Melbourne, 8001 Australia

**Keywords:** Expensive optimization problem, evolutionary computation, evolutionary algorithm, swarm intelligence, particle swarm optimization, differential evolution

## Abstract

Expensive optimization problem (EOP) widely exists in various significant real-world applications. However, EOP requires expensive or even unaffordable costs for evaluating candidate solutions, which is expensive for the algorithm to find a satisfactory solution. Moreover, due to the fast-growing application demands in the economy and society, such as the emergence of the smart cities, the internet of things, and the big data era, solving EOP more efficiently has become increasingly essential in various fields, which poses great challenges on the problem-solving ability of optimization approach for EOP. Among various optimization approaches, evolutionary computation (EC) is a promising global optimization tool widely used for solving EOP efficiently in the past decades. Given the fruitful advancements of EC for EOP, it is essential to review these advancements in order to synthesize and give previous research experiences and references to aid the development of relevant research fields and real-world applications. Motivated by this, this paper aims to provide a comprehensive survey to show why and how EC can solve EOP efficiently. For this aim, this paper firstly analyzes the total optimization cost of EC in solving EOP. Then, based on the analysis, three promising research directions are pointed out for solving EOP, which are problem approximation and substitution, algorithm design and enhancement, and parallel and distributed computation. Note that, to the best of our knowledge, this paper is the first that outlines the possible directions for efficiently solving EOP by analyzing the total expensive cost. Based on this, existing works are reviewed comprehensively via a taxonomy with four parts, including the above three research directions and the real-world application part. Moreover, some future research directions are also discussed in this paper. It is believed that such a survey can attract attention, encourage discussions, and stimulate new EC research ideas for solving EOP and related real-world applications more efficiently.

### 1 Introduction

Expensive optimization problem (EOP)[1−3] refers to the problem that requires expensive or even unaffordable costs to evaluate candidate solutions, which widely exist in many significant real-world applications. For example, the deep learning-based AlphaFold2 for predicting protein structure is considered worldwide as a potential approach to a 50-year-old grand challenge in biology[4, 5]. But the training of AlphaFold2 (i.e., optimizing the large-scale network parameters) requires 128 TPUv3 cores (i.e., the third generation of tensor processor unit for AI/deep learning) together with several weeks, where a TPUv3 can have much larger computational abilities than many supercomputers[6]. That is, the parameter optimization of AlphaFold2 is very computationally expensive and will be unaffordable for many researchers. Moreover, the "expensive cost" is a relative concept rather than an absolute concept in many real-world problems. For instance, when encountering the emergencies like epidemics outbreaks or natural disasters, transportation and dispatching can be urgent for supporting daily operations and saving lives, where the time cost of optimization in normal situations will become too expensive to accept at this time[7]. Therefore, with the progress of real-world society and the emerging issues like the smart cities, the internet of things, and the big data era, solving EOPs more efficiently has become increasingly essential for prospering various fields.

However, due to the expensive cost of evaluating candidate solutions, EOP is difficult for optimization algorithms to search for a satisfactory solution[8]. For this aim, evolutionary computation (EC) has been widely adopted to solve EOPs, leading to a fast-growing research field[8−11]. In general, EC is a type of optimization methodology that is inspired by biological evolution and live

Review
Manuscript received August 3, 2021; accepted October 9, 2021
Recommended by Associate Editor Min-Ling Zhang
Colored figures are available in the online version at https://link.springer.com/journal/11633
© The Author(s) 2022

organism characteristics[12, 13]. Commonly seen EC algorithms include evolutionary algorithm (EA) like genetic algorithm (GA)[14] and differential evolution (DE)[15−17], and swarm intelligence algorithms like particle swarm optimization (PSO)[18−22] and ant colony optimization (ACO)[23−25]. With the idea of "survival of the fittest" from natural evolution, EC algorithms generate new individuals via corresponding evolutionary operators and select individuals with better fitness as a new population into the next generation. Based on this, EC algorithms can find a satisfactory solution efficiently without the need for gradient information, which is very suitable for solving real-world problems[26].

To date, various researches into EC for EOP have been conducted and achieved considerable success. However, the work of EC for EOP is still dispersed in the literature and remains to be consolidated in a systematic manner. Therefore, given the rapid and important advancements of EC for EOP, it is essential to review these advancements in order to synthesize and give previous research findings and references to help develop relevant research fields. For this purpose, this paper attempts to provide a systematic and comprehensive survey to completely review and analyze how to enable and develop EC algorithms for tackling difficult EOPs efficiently. In addition to present the review more concisely and clearly, this paper selects and cites related work by considering their sources, publication years, impact, and the cover of different aspects of the topic surveyed in this paper. Overall, the main contributions of this paper are given as follows.

Firstly, this paper mathematically analyzes the total expensive cost of using EC for solving EOPs. Then, based on the analysis, this paper further gives three directions for reducing the total cost, as shown in Section 2 later. Note that, to the best of our knowledge, this paper is the first that derives the potential research directions by analyzing the total expensive cost of using EC for solving EOPs.

Secondly, a systematic taxonomy is introduced to systematically and structurally review the existing works according to their efforts in the above-pointed directions for solving EOPs efficiently. Although there exist some survey papers about EC for EOP in the literature[1−3], our survey is based on the above novel taxonomy. It, therefore, can help better understand why and how existing works can be helpful in solving EOPs, as well as providing inspiration for future researches and studies.

Thirdly, this paper explores and discusses some future research areas and open problems related to the use of EC to tackle EOPs. This can encourage and support the broadening and deepening of research in the related fields.

The remainder of this paper is arranged as follows. Section 2 provides the preliminaries, including the definition of EOP, a brief introduction to the EC, the analysis of total cost for using EC to solve EOPs, and the novel taxonomy. Sections 3−6 review the research work systematically according to the taxonomy. Section 7 proposes and discusses some future research directions, while Section 8 gives the conclusions finally.

### 2 Preliminaries

#### 2.1 Expensive optimization problem and relevant optimization problems

EOP is the same as other optimization problems except that the evaluation of candidate solutions is computationally/financially expensive to perform. Note that the evaluation can contain various aspects including the objective value, constraint violation degree, and robustness of candidate solutions. According to the problem characteristics and application needs, EOP can be extended to the corresponding complex problem[26, 27].

Moreover, the concept of EOP is usually mentioned together with some similar problem concepts, including data-driven optimization problem, surrogate-assisted optimization problem, and large-scale optimization problem. The relationship between EOP and these problems can be presented as Fig. 1, and is described as follows.

**Surrogate-assisted optimization problem**[8]. Surrogate-assisted optimization problem refers to the problem that uses surrogates to assist the optimization. Often, the reason for using surrogates is that the fitness or constraint evaluation of candidate solutions is expensive or difficult to access. Based on this, most surrogate-assisted optimization problems are EOP. In fact, surrogates are also widely used to solve EOPs in the literature, where the related contents will be described later in Section 3.

**Data-driven optimization problem**[9, 10]. The data-driven optimization problem is a type of real-world optimization problem that only data acquired from simulations, physical experiments, or daily life, can be used to evaluate the objectives and constraints of candidate solutions, because computationally cheap functions do not exist for this problem. Therefore, many data-driven optimization problems can also be considered as a special kind of EOP.

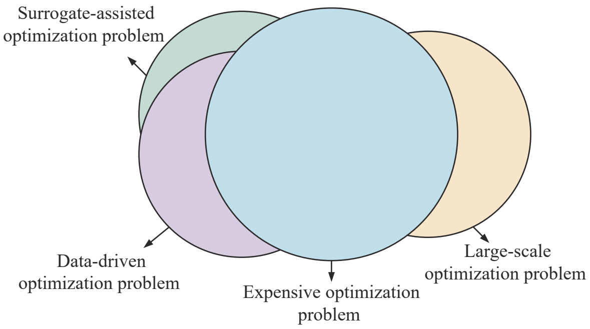

Fig. 1 Relationship between expensive optimization problem and some relevant optimization problems

**Large-scale optimization problem**[28]. Large-scale optimization problems focus on the large-scale challenges that make optimization difficult. The challenges can include large search space, high variable dimension, and the need to process a large amount of data for evaluating solutions. As a result, the large-scale characteristic often makes the related calculation (e.g., fitness evaluation) expensive, and therefore many large-scale optimization problems can be regarded as EOPs[29, 30].

### 2.2 Evolutionary computation

As mentioned earlier in the Introduction part, EC algorithms evolve better solutions generation by generation. In general, the flowchart of EC can be presented as Fig. 2, which includes initialization, fitness evaluation (FE), solution evolution with evolution operators, and selection. Moreover, the solution evolution, FE, and selection will be iteratively performed to generate and select better solutions from the previous generation to the next generation, so as to obtain the optimal or satisfactory solution. Therefore, the main computational cost of EC lies in performing evolutionary operators, FE, and selection, especially when the problem is an EOP.

### 2.3 Analysis of total optimization cost

As the key characteristic of EOP is the expensive evaluation, the total cost of using EC for solving the EOP can be defined as

$$Total\_cost = O(N) \times O(C) = O(N \times C) \tag{1}$$

where $O(C)$ is the average cost of every expensive evaluation, $O(N)$ indicates the time complexity of an EC algorithm for solving the problem with respect to the number of evaluations (i.e., the total number of required evaluations for finding a satisfactory solution). Specifically, if $O(C)$ refers to the computational time cost, then (1) can be further rewritten as

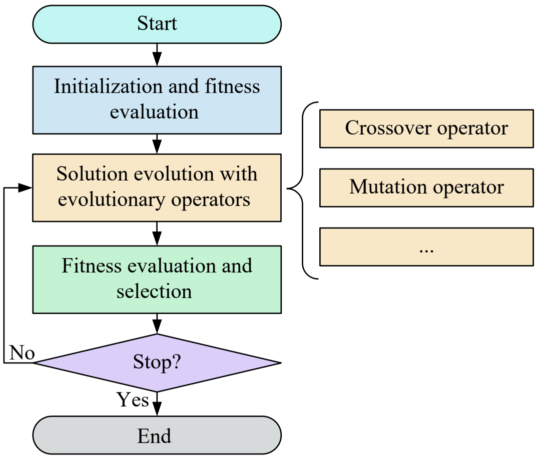

Fig. 2 General flowchart of evolutionary computation

$$Total\_cost = \frac{O(N) \times O(C)}{P} = O(\frac{N \times C}{P}) \tag{2}$$

where $P$ ($P \geq 1$) is the acceleration of optimization provided by parallel and distributed computation techniques.

As mentioned before, the key issue in EOP is the expensive fitness and/or constraint evaluation, i.e., $O(C)$ in (1) and (2) will be very large, which lead to an unaffordable *Total_cost*. However, (1) and (2) also indicate that efforts can be made in three directions to solve the EOP more efficiently. These three directions are decreasing $O(C)$, decreasing $O(N)$, and increasing $O(P)$. In fact, existing works for EOP have been proposed and studied implicitly along with these three directions, which can be summarized as using problem approximation and substitution to reduce evaluation cost to decrease $O(C)$, designing advanced EC algorithms to improve search efficiency to reduce $O(N)$, and using parallel and distributed computation to accelerate the optimization by increasing $O(P)$, as illustrated in Fig. 3.

### 2.4 Taxonomy

Based on the above contents and Fig. 3, the systematic taxonomy of existing research works can include three parts: problem approximation and substitution, algorithm design and enhancement, and parallel and distributed computation. Besides, as EOP arises from real-world applications, reported case studies and benchmark problems derived from real-world applications should also be surveyed. Therefore, the taxonomy of this paper includes four major parts, as shown in Fig. 4. Specifically, the first part (i.e., problem approximation and substitution) includes problem simplification, fitness approximation, constraint approximation, and multi-fidelity substitution; the second part (i.e., algorithm design and enhancement) introduces optimization framework and paradigm, novel operators, fitness inheritance, and hybrid algorithms and configurations; the third part (i.e., parallel and distributed computation) considers accelerations for approximation and optimizations; and the fourth part (i.e., real-world applications) is about the real-world

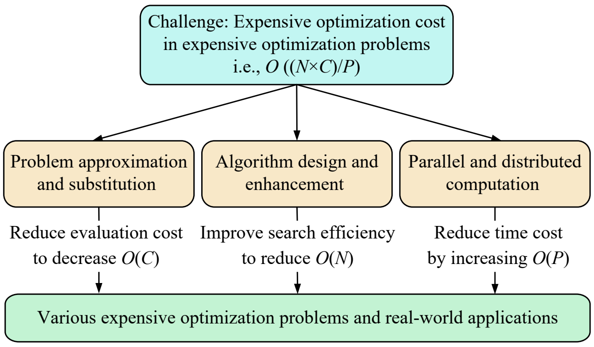

Fig. 3 Three directions to reduce expensive optimization costs

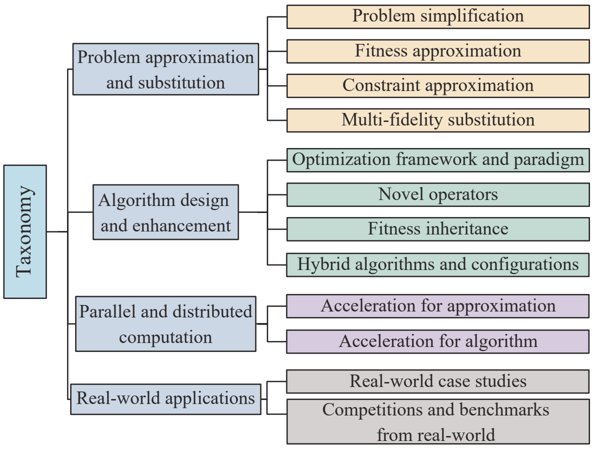

Fig. 4 An illustration of the taxonomy case studies and competitions and benchmarks from real-world applications.

## 3 Problem approximation and substitution

In many optimization problems, mathematical or exact objective/constraint functions for evaluating solutions may not exist. In such situations, candidate solutions can only be evaluated by computationally expensive numerical simulations or physical experiments, e.g., wind tunnel experiments. This poses great challenges on EC, because most EC algorithms are based on FEs for evolutionary optimization. In order to address this issue and reduce the optimization difficulty, approximation methods have been widely researched[8, 9]. Generally speaking, existing approximation methods can be mainly classified into four categories, i.e., problem simplification, fitness approximation, constraint approximation, and multi-fidelity substitution.

### 3.1 Problem simplification

Problem simplification is a straightforward yet efficient approach when the original optimization problems are computationally expensive. Problem simplification aims to simplify the original EOP to be a problem model that is more computationally efficient to access, so as to relieve the expensive computational burden. For example, to replace the original expensive simulation for the dispatching rules design problem, Nguyen et al.[11] developed a simplified simulation model with two proposed strategies. The first strategy eliminates the warmup and running time in simulations, whereas the second strategy minimizes the search space, e.g., by reducing the number of operations and machines for each job. The experimental results reveal that by combining the two strategies, a simplified but accurate enough simulation model can be developed with significantly less computational cost than the original and complete simulation evaluation. Furthermore, He et al.[31] formulated the expensive ratio error estimation problem for the voltage transformer into a relatively simple and cheaper problem. Then, eight representative multi-objective evolutionary algorithms were used to analyze and solve the problem. Voutchkov et al.[32] suggested a simplified mathematical model for solving sequential combinatorial finite element problems, which would minimize the expensive computational cost considerably. Besides, in some real-world optimization problems, there have been many FEs with different computational costs for simplifying the specific real-world EOP. For example, 2-D and 3-D computational fluid dynamics (CFD) simulation can be used to replace the real-world wind tunnel experiment during the optimization of the aerodynamic structure, where the 2-D and 3-D simulations are significantly more computationally cheap[33].

### 3.2 Fitness approximation

Unlike the problem simplification methods that simplify the original problem, the fitness approximation directly approximates or predicts the fitness value of candidate solutions, so as to reduce the expensive cost of performing the expensive FE. Mathematically, for a continuous function, the relationship between the fitness of $x$ predicted by the approximated fitness function, say $f(x)$, and that given by the real fitness function, say $F(x)$, can be expressed as

$$F(x) = f(x) + \varepsilon(x) \tag{3}$$

where $\varepsilon(x)$ represents the approximation error of $f$ on $x$.

In general, fitness approximation methods approximate the objective fitness function based on evaluated data, i.e., the solutions evaluated by the original expensive FE. Based on this, the approximated objective function (also called surrogate model) will be employed to substitute the original FE to evaluate un-evaluated candidate solutions[9]. Therefore, fitness approximation-based optimization is often referred as data-driven or surrogate-assisted optimization. In such an end-to-end approach, the fitness approximation only needs to focus on the relationship between candidate solutions and their real fitness. That is, the fitness approximation approach has a great generalization ability and can be used to solve a variety of EOPs. As a result, many fitness approximation methods have been investigated and proposed[9–12].

Generally speaking, the fitness approximation mainly includes three procedures: data processing, surrogate model building, and model update and management. In order to approximate the fitness function and build surrogates accurately, various model building methods can be used, including traditional interpolation methods, such as polynomial regression model[34, 35], and machine learning techniques, such as the Kriging model[36–38], artificial neural networks[39, 40], radial basis function neural networks[41–44], and random forest[45–47]. To better show the application scope of different surrogates, the comparison of some commonly seen surrogates is presented in Table 1.

Moreover, various fitness approximation methods have also been examined using effective machine learning strategies. For example, for constructing accurate surrogates, Wang et al.[48] proposed to use the active learning strategy to construct a committee-based surrogate ensemble for solving EOPs, which is shown to be effective. As for offline data-driven optimization problems, Wang et al.[49] investigated an ensemble learning-based surrogate management strategy that adaptively selects a small but diverse selection of surrogates to improve the approximation accuracy. To enhance the surrogate robustness and accuracy, Wei et al.[50] suggested a gradient boosting classifier, which can generate promising results with a limited training dataset. Considering that the evaluated data might not be enough for fitness approximation, Li et al.[51] proposed a localized data generation (LDG) method with a boosting learning method to obtain a better surrogate model, which can automatically improve both model accuracy and data quantity according to the targeted problem. Besides, Li et al.[52] combined a novel diverse surrogate generation method and selective ensemble method to design the perturbation-based ensemble surrogates (PES) for data-driven optimization, resulting in a larger training data amount, better data utilization, and enhanced surrogate accuracy in the proposed algorithm. These are the recent boosting data-driven EA with LDG (BDDEA-LDG) and data-driven EA with PEs (DDEA-PES) algorithms.

Besides finding a general approximated function via machine learning methods, fitness imitation has also been researched for evaluating individuals. Generally speaking, fitness imitation predicts the fitness of a new individual based on evaluated individuals that are related to that individual. For example, Salami and Hendtlass[53] suggested a fitness imitation method in which the new individual's fitness is the weighted sum of the evaluated individuals' fitness. Sun et al.[54] proposed a fitness imitation strategy that estimates the fitness of the targeted individual based on the distance between it and other evaluated individuals. Furthermore, Tian et al.[55] presented a Gaussian similarity measurement to better estimate individual fitness. Besides, Kim and Cho[56] split the population into different clusters and then estimated the fitness of the majority individual in each cluster based on the evaluated individual in the corresponding cluster. Similarly, Jin and Sendhoff[57] put forward a cluster-based method to decrease the FE, in which the individual closest to the cluster center is accessed using real fitness data while the others are accessed via estimation.

### 3.3 Constraint approximation

Real-world optimization problems usually contain constraints that need to be satisfied. Moreover, the constraint functions can be expensive to access during the FE. Therefore, many studies have been conducted to handle the expensive constraint functions via constraint approximation[58]. Similar to the fitness approximation described above in Section 3.2, expensive constraint functions can also be approximated via an end-to-end fashion, so as to reduce the expensive computational cost in FE. Consequently, methods mentioned in Section 3.2 for fitness approximation can also be used for constraint approximation, with one model for all constraints or multiple models for multiple constraints separately[59]. When compared with fitness approximation, the distinct and challenging issue in constraint approximation is how to handle the approximated constraints, which can be categorized into two main categories: full constraints-based and partial constraints-based handling techniques.

The first category handles all constraints via approximation. After approximating the constraint function, EC algorithms can use constraint handling techniques to generate feasible solutions. In general, commonly seen constraint handling techniques involve penalty method[60], repair method[61], multi-objective method[62], and epsilon level comparison method[63]. For example, Li and Zhang[60] proposed a method termed multiple penalties and multiple local surrogates for solving expensive constrained problems. The method identifies some subproblems and constructs local surrogates to optimize these subproblems in every generation. Each subproblem penalizes the constraints in the original problem with a different penalty coefficient and different local search subregions. By doing so, the method can approach the optimal solution from different directions based on different constraint influences, which only requires a low computational overhead for building corresponding local surrogates. Habib et al.[63] suggested a multiple surrogates-assisted decomposition-based evolutionary algorithm for solving

Table 1 Comparisons of commonly seen surrogates

| Surrogate | Main applicable scope | Characteristic |
|---|---|---|
| Gaussian process (Kriging model) | Continuous problem | High accuracy with the estimation of prediction uncertainty, but with a high computational burden |
| Artificial neural network | Continuous problem | High accuracy, long training time |
| Radial basis function neural network | Continuous problem | Highly nonlinear approximation ability, low computational burden |
| Random forest | Discrete and combinational problem | Less tuning parameters, suitable for discrete input |

the expensive constrained problem, where an epsilon level comparison method is used to accept some individuals who violate constraints slightly to enhance the evolutionary search.

In contrast to considering all constraints, the partial constraints-based handling technique aims to utilize some of the representative constraints to replace all constraints without decreasing the quality of final solutions, so as to reduce the expensive cost of accessing unnecessary constraint evaluations. In order to achieve this, a straightforward idea is to use the constraint functions one by one and stop immediately until the solution violates the constraints, which is termed the "evaluate till you violate" approach[64]. Following this, some studies have been undertaken to find suitable sequences for performing constraint evaluations one by one, e.g., the sequence in a ring topology[65] and flexible sequence for different sub-populations[66, 67], so as to minimize the unnecessary evaluation costs. Moreover, Rahi et al.[68] proposed to learn the suitable sequence for performing constraint evaluations during the optimization, which has obtained better results than existing methods.

#### 3.4 Multi-fidelity substitution

In many real-world optimization problems, the fidelity (i.e., accuracy level) and computational cost of the FE could be modified with various settings and configurations such as simulation time[69]. As a result, there is a tradeoff between the fidelity level of the evaluation and the corresponding computational cost. Moreover, approximation methods with different fidelity or accuracy level could be cooperated to obtain a better final model. Based on the above, multi-fidelity substitution looks at ways to get a multi-fidelity evaluation model with a better balance between evaluation fidelity and computational cost to substitute the expensive FE. For example, Alexandrov et al.[70] put forward a variable-fidelity model for meshes of various refinement based on Euler analysis. Zheng et al.[71] developed a variable fidelity optimization (VFO) framework to achieve high-speed and high-fidelity optimization. Lim et al.[72] developed a dynamic fidelity computational model for FE, in which the fidelity of the computational model grows as the evolution progresses. Koziel[73] investigated a multi-fidelity optimization in which the computational model′s fidelity level can be adaptively modified. With simulation-based FE of different accuracy scales, Wu et al.[74] developed a scale-adaptive FE (SAFE) approach for the crowdshipping scheduling application problem, which can strike a better balance between solution accuracy and computational cost. Li et al.[75] proposed a surrogate-assisted multi-level evaluation method to reduce the expensive computational cost in optimizing the CNN hyperparameters. In this method, two levels of evaluation, i.e., surrogate-based and training-based FE, are cooperated to balance the optimization accuracy and computational cost, which has shown great efficiency on real-world data.

Furthermore, some studies adopted a number of Kriging models to approximate the FE with varying degrees of validity[76−78]. In such methods, the correlations between multiple Kriging models can be estimated using the bridge function of Kriging models to help the selection of different evaluation models[79].

In contrast to the selection for multi-fidelity models, Smith et al.[80] explored how to produce models with both high accuracy and low complexity, and suggested a multi-objective algorithm to address this issue. Sun et al.[81] designed a multi-fidelity optimization for the sheet metal forming process, in which low-fidelity models are used to approximate the high-fidelity models. Li et al.[82] used a high-fidelity model to break the problem down into several sub-problems and then adopted the low-fidelity model to approximate each sub-problem. In addition, Sun et al.[43] also coupled the fitness approximation and evolutionary approximation with different fidelities for high dimensional expensive problems, which have proved to be beneficial. Besides, Li et al.[77] designed a three-level surrogate model method for solving expensive optimization, where the three levels surrogate are utilized to guide the optimization for global search, subregion search, and local exploitation search, respectively.

#### 3.5 Comparisons and discussions

This part gives a comparison and discussion on state-of-the-art problem approximation methods. According to the category in problem approximation methods for EOP, Table 2 lists and compares some representative works in recent three years, i.e., those published in IEEE Transactions from 2019 to 2021. In Table 2, three observations can be obtained. First, there is a lack of work for problem simplification in IEEE Transactions in recent years, which suggests that more research focuses are given to the other three categories. Second, the majority of these works are for multi-objective/many-objective and constrained problems. This may be due to that real-world application problems often have multiple objectives and constraints. Third, most methods consider fitness approximation with surrogates to learn a computationally efficient model to replace the original expensive FE. The reasons might be that with the development of artificial intelligence, end-to-end learning-based approximation methods have become more accurate, efficient, and convenient for researchers to use in these years.

### 4 Algorithm design and enhancement

This section reviews the methods for solving EOP via efficient algorithm design and enhancement. In fact, besides EOP, it is also very significant when evaluating an EC algorithm for solving non-expensive problems, which results in many studies for improving algorithm effi-

Table 2 Comparisons of some recent works with different problem approximation methods

| Category | Recent works | Problem features | Approximation method | Year | Publication |
|---|---|---|---|---|---|
| Fitness approximation | MGP-SLPSO[36] | Expensive, high-dimensional | Multi-objective surrogate considering approximation accuracy and uncertainty | 2019 | *IEEE Transactions on Evolutionary Computation* |
| | KTA2[37] | Expensive, many-objective | Influential point-insensitive model with an adaptive infill criterion | 2021 | *IEEE Transactions on Cybernetics* |
| | GCS-MOE[38] | Expensive, many-objective | Multi-task surrogate to approximate subproblems | 2019 | *IEEE Transactions on Cybernetics* |
| | MTCNP[39] | Expensive, multi-task | Surrogate-assisted multi-task learning | 2020 | *IEEE Transactions on Evolutionary Computation* |
| | E2EPP[47] | Expensive optimization of the hyperparameters of deep learning models | Random forests with a selective ensemble strategy | 2020 | *IEEE Transactions on Evolutionary Computation* |
| | DDEA-SE[49] | Single-objective, small data-driven | Selective surrogate ensembles of radial basis function neural networks | 2019 | *IEEE Transactions on Evolutionary Computation* |
| | CA-LLSO[50] | Expensive, online data-driven, single-objective | Gradient boosting of weak models to obtain a powerful model | 2021 | *IEEE Transactions on Evolutionary Computation* |
| | BDDEA-LDG[51] / DDEA-PES[52] | Single-objective, small data-driven | Surrogate ensembles with data generation | 2020/ 2021 | *IEEE Transactions on Evolutionary Computation/ IEEE Transactions on Evolutionary Computation Cybernetics* |
| | HSMEA[63] | Expensive, multi-/ many-objective | Approximating each objective function | 2019 | *IEEE Transactions on Evolutionary Computation* |
| | TLRBF[77] | Expensive, single-objective | Three-level surrogate model | 2021 | *IEEE Transactions on Cybernetics* |
| | DSCPSO-EMM[83] | Expensive, multi-modal | Modal-guided dual-layer cooperative surrogate model | 2021 | *IEEE Transactions on Evolutionary Computation* |
| | MiSACO[84] | Expensive, mixed-variable | Combination of surrogates for mixed-variable approximation | 2021 | *IEEE Transactions on Cybernetics* |
| | SGA[85] | Expensive, high-dimensional | Approximated surrogates for local search and solution update | 2020 | *IEEE Transactions on Evolutionary Computation* |
| Constraint approximation | RF-CMOCO[45] | Data-driven, constrained, multi-objective, combinatorial | Approximating objective and constraint functions separately | 2020 | *IEEE Transactions on Evolutionary Computation* |
| | MPMLS[60] | Expensive, constrained | Penalizing the constraints in the original problem | 2021 | *IEEE Transactions on Evolutionary Computation* |
| | GLoSADE[61] | Expensive, constrained | Using surrogates to refine solutions that violate constraints | 2019 | *IEEE Transactions on Cybernetics* |
| | SParEA[68] | Expensive, constrained | Using partial evaluation to substitute full evaluations | 2021 | *IEEE Transactions on Evolutionary Computation* |
| Multi-fidelity substitution | SAFE[74] | Expensive, single-objective | A novel scale-adaptive fitness evaluation method | 2021 | *IEEE Transactions on Evolutionary Computation* |
| | SHEDA[75] | Expensive, mixed-variable | A surrogate-assisted multi-level evaluation method | 2021 | *IEEE Transactions on Neural Networks and Learning System* |

ciency via the algorithm design and enhancement. As a result, numerous existing works to improve algorithm efficiency for non-expensive problems can also be adopted to solve EOPs. However, to concentrate on the topic of this survey, this section will only review related work for improving the efficiency of EC for EOP, which are categorized into four parts, including optimization framework and paradigm, novel operators, fitness inheritance, and hybrid algorithms and configurations.

### 4.1 Optimization framework and paradigm

As various EOPs have different characteristics, using suitable optimization framework and paradigm can solve the targeted problem in a more efficient way. For solving various EOPs, commonly seen optimization frameworks include multi-population/multi-swarm evolution[86, 87], co-evolution[88–90], decomposition-based evolution[63, 91], while widely used optimization paradigms involve single-objective optimization[51], multi-objective/many-objective optimization[92–95], constrained optimization[96], multimodal optimization[83, 97, 98], mix-variable optimization[84, 99], and multi-task optimization[100, 101].

Moreover, with proper problem transformation and reformulation methods, efficient optimization paradigms can be utilized to solve a variety of problems. For example, Han et al.[102] transferred a multi-objective optimization problem into a number of scalar subproblems and utilized infill-sampling criteria and constraint handling method to solve the subproblems, where real-world simulation-based engineering design optimization problems have verified the effectiveness of the proposed approach. Liao et al.[103] treated the multiple surrogate-assisted optimization processes for solving a computational EOP as a multitask optimization procedure, and then proposed to use multi-tasking optimization methods to accelerate the convergence. Experimental results have validated that treating multiple surrogate-assisted optimizations as a multitask optimization is effective on twelve widely used benchmark problems of up to 200 dimensions. However, although various optimization frameworks and paradigms have obtained great results for EOP, it could be better to choose the suitable framework and paradigms based on the prior knowledge about the problem characteristics and features, so as to obtain more satisfactory results.

### 4.2 Novel operators

Designing novel evolutionary operators can be helpful to accelerate the convergence and enhance the optimization accuracy of algorithms, so as to solve EOPs more efficiently. For example, Cai et al.[104] proposed a novel mutation operator that integrates the best solution indicated by different surrogates, so as to guide the mutation direction of the populations, where the effectiveness of the proposed operator is demonstrated in the experiments on high dimensional EOPs with dimensions up to 200. Gu et al.[105] presented an adaptive probability operator-based stochastic ranking strategy that enhances solution quality by considering both convergence and diversity. The proposed method is effective for constrained and multi-objective EOPs according to the numerical studies in the paper. Zhang et al.[106] adopted a batched constrained decomposition method with grids to balance the population convergence and diversity, so as to conduct better the batched sampling for solving the multi-objective EOP, which is shown effectiveness on difficult test problems. Besides, various methods have also been studied to improve the algorithm efficiency[85, 107–109].

### 4.3 Fitness inheritance

Fitness inheritance is similar to the fitness approximation mentioned in Section 3.2 in that both of them attempt to reduce the need for expensive FEs by estimating some individual fitness. However, they are different in that the fitness inheritance is designed based on the individual relationship given by the evolutionary operator[3], while fitness approximation does not consider the evolutionary operator in EC. In general, fitness inheritance calculates the fitness of candidate individuals based on other related individuals during the evolution, such as the parents in crossover operators. For this, Smith et al.[110] demonstrated the theoretical and actual efficiency of fitness inheritance for GA. Furthermore, Chen et al.[111] suggested a fitness inheritance method to accelerate the algorithms, while Zheng et al.[112] used a fitness inheritance method in GA to tackle a design problem on vector quantization codebooks. In addition, Sastry et al.[113] provided analyses of the fitness inheritance in general EA. Although the fitness inheritance effectively reduces the need for FEs, it has a similar usage when compared with the methods for problem approximation and substitution (as mentioned in Section 3). Therefore, it might be helpful to combine the idea of fitness inheritance with methods for problem approximation and substitution to solve EOPs more efficiently.

### 4.4 Hybrid algorithms and configurations

Given that different algorithms may be suitable for different EOPs, hybrid algorithms have also been considered for solving various EOPs more efficiently. Zhan et al.[114] proposed an algorithm called Cloudde that integrates four populations with different configurations by an efficient resource allocation, which has shown significant efficiency in the design of power electronic circuits where the FE is very computationally expensive. Sun et al.[43] suggested a cooperative algorithm that hybrids a PSO algorithm for local search and a social learning PSO algorithm for exploration, so as to improve search efficiency. The proposed algorithm is shown to be efficient in solving high-dimensional EOPs. Zhen et al.[115] proposed data-driven evolutionary sampling optimization framework that combines two evolutionary sampling strategies and employs one of them in different evolutionary stages to effectively balance the global and local searches. In addition, other hybrid algorithms and configurations have also been studied and shown effective[116–118].

### 4.5 Comparisons and discussions

This part gives a comparison and discussion on the algorithm design and enhancement of the state-of-the-art works. According to the category in algorithm design and enhancement for EOP, Table 3 lists and compares some recent representative works in the recent three years, i.e., those published in IEEE Transactions from 2019 to 2021. Table 3 offers four observations. First, the works listed in Tables 2 and 3 have many overlaps. This indicates that both efficient problem approximations and powerful op-

Table 3 Comparisons of some recent works with different algorithm designs and enhancements

| Category | Recent works | Problem features | Algorithm design and enhancement | Year | Publication |
|---|---|---|---|---|---|
| Optimization framework and paradigm | MPMLS[60] | Expensive, constrained | Constrained optimization by assigning individuals to optimize penalty sub-problems | 2021 | *IEEE Transactions on Evolutionary Computation* |
| | HSMEA[63] | Expensive, multi-/many-objective | Decomposition-based multi-objective optimization with a proposed local search operator | 2019 | *IEEE Transactions on Evolutionary Computation* |
| | CSEA[95] | Expensive, many-objective | Many-objective optimization framework based on a proposed dominance relationship prediction | 2019 | *IEEE Transactions on Evolutionary Computation* |
| | DSCPSO-EMM[83] | Expensive, multi-modal | Multi-modal optimization based on a dual-population cooperative particle swarm optimizer and a hybrid strategy of clustering and peak-valley | 2021 | *IEEE Transactions on Evolutionary Computation* |
| | MiSACO[84] | Expensive, mixed-variable | Mixed-variable optimization based an ant colony optimization with three proposed selection operators | 2021 | *IEEE Transactions on Cybernetics* |
| | G-MFEA[100] | Expensive, multi-task | Multi-task optimization with two strategies for decision variable translation and shuffling | 2019 | *IEEE Transactions on Evolutionary Computation* |
| Novel operators | AE-CNN+E2EPP[47] | Computationally expensive evaluation of the hyperparameters of deep learning models | GA with block-based and variable-length encoding scheme | 2020 | *IEEE Transactions on Evolutionary Computation* |
| | CA-LLSO[50] | Expensive, online data-driven, single-objective | Level-based learning swarm optimizer with an exploitation strategy | 2021 | *IEEE Transactions on Evolutionary Computation* |
| | GLoSADE[61] | Expensive, constrained | DE with two mutations strategies for global and local search | 2019 | *IEEE Transactions on Cybernetics* |
| | SAFE[74] | Expensive, single-objective | Cooperative of evaluation methods with different scales and a neighbor best-based fitness evaluation strategy | 2021 | *IEEE Transactions on Evolutionary Computation* |
| | SHEDA[75] | Expensive, mixed-variable | Surrogate-assisted hybrid-model estimation of distribution algorithm with a novel initialization method and an adaptive model learning method | 2021 | *IEEE Transactions on Neural Networks and Learning System* |
| | SGA[85] | Expensive, high-dimensional | GA with a crossover based on predicted parents and a local search operator | 2020 | *IEEE Transactions on Evolutionary Computation* |

timization algorithms are essential in solving EOPs efficiently. Second, the listed works are in the category of optimization framework and paradigm or novel operators, which indicates the recent research focus and direction in algorithm design for EOP. Third, enhanced algorithms are mainly the variants of GA, DE, and PSO. This may be because that these three algorithms are effective and efficient in solving various complex optimization problems. Fourth, as shown in Table 3, different EC algorithms are used for different problems, suggesting that different EC algorithms may be suitable for different EOPs.

### 5 Parallel and distributed computation

When evaluating candidate solutions is computationally expensive, parallel and distributed techniques can be used to accelerate the optimization and reduce the computational time cost. Existing related works can be roughly classified into two categories, i.e., acceleration for approximation and acceleration for the optimization process, which are described as follows.

#### 5.1 Acceleration for approximation

Acceleration for approximation refers to the methods that incorporate parallel and distributed techniques with simulation and surrogate models. In an aerodynamic shape optimization problem, Karakasis et al.[119] suggested a distributed EA with surrogates to help reduce the expensive computational time cost of invoking unnecessary computational fluid dynamics. The algorithm will divide the population into some sub-populations and evolve them with surrogates simultaneously. The best solution in each sub-population will be migrated to other sub-populations regularly to improve the optimization. The results in the experimental studies reveal that combining surrogates and distributed methods yields the highest computational efficiency in term of the time cost. Furthermore, Akinsolu et al.[120] suggested a parallel model to improve the parallelization of surrogate-assisted EA. To be specific, the proposed algorithm selects various solutions with potential fitness together for the computationally expensive FE. During this, the expensive FE of each selected solution can be done simultaneously, so as to reduce the expensive computational time cost.

Despite the above, the algorithm parallelization can be further improved if there are multi-fidelity or multi-level surrogates when solving EOPs. For example, Karakasis et al.[121] put forward a hierarchical distributed EA for solving the expensive shape optimization problem, where each sub-population uses a surrogate with different accuracy levels and computational time costs. During the distributed evolution of sub-populations, better solutions will be reallocated to sub-populations that use a more expensive but also more accurate surrogate, while worse solutions will be reallocated to sub-populations with a less accurate but cheaper surrogate, so as to fully utilize the computational budget. In addition, Sun et al.[122] presented an asynchronous parallel surrogate optimization algorithm, which is based on the ensemble surrogate model and the stochastic response surface method. The algorithm uses parallel computing techniques to speed up both the weights update of ensemble surrogates and parameter optimization, which has proven to be efficient for solving EOPs.

### 5.2 Acceleration for optimization

Despite the approximation, many parallel and distributed methods have been investigated to speed up the optimization process of EC algorithms, so as to solve EOPs more efficiently[123]. For example, a parallel DE is proposed by Liu et al.[124] for solving the power electronic circuit optimization problem with simulation-based time-consuming FEs. The parallel DE can assign candidate solutions to diverse resources for parallel FEs based on distributed cloud computing resources, and thereby reducing the expensive time cost. The experimental results showed that the parallel DE could significantly speed up the evolutionary search process. Knowing that the computational resources may have varying workloads and computing capabilities, Ma et al.[125] developed a distributed DE with a load balance technique for efficiently and correctly allocating computational FE tasks to various resources. This method considers and calculates the load information of each resource for executing dynamic resource allocations, which allows the topology between individuals and resources to change adaptively for the better usage of the concurrent computational resources. Different from just assessing the load information of resources, Zhan et al.[114] proposed a distributed cloud-ready differential evolution (Cloudde), which considers both the resource workload and the computational cost of all FE tasks. The Cloudde estimates the suitable number of FEs assigned to each processing node based on the corresponding resource workloads and computational burdens, and then allocates the individuals for FEs accordingly, which has proven to be very efficient in both numerical simulation and a real-world case study on power electronic circuit design application. Similarly, to solve complex EOPs efficiently, Zhan et al.[126] developed an adaptive distributed DE, where three populations (for exploration, exploitation, and balance) co-evolve concurrently using the master-slave multi-population distributed framework. Experimental results on extensive benchmark problems and real-world problems have shown the great search efficiency of the proposed distributed DE. Besides, for the expensive neural-network-based controller optimization in a power electronic circuit, Liu et al.[127] investigated a resource-aware method to utilize distributed resources more efficiently by adaptively assigning individuals to resources depending on the real-time performance of the resources, which can significantly reduce the expensive computational time cost. Besides, Li et al.[128] proposed a generational-level parallelism to develop a pipeline-based parallel PSO algorithm, which has obtained promising speedup on various problems with different time delays in every FE.

In addition, as shown in Fig. 1, optimization problems with large-scale characteristics are often also EOPs. Moreover, the large-scale characteristics of some EOPs, such as large search space, high variable dimension, and the large quantity of data, often result in more expensive computational costs for optimization. When solving such large-scale optimization problems and/or large-scale EOPs, distributed methods can help improve algorithm efficiency to reduce the expensive time cost. For example, Ge et al.[129] suggested a distributed DE with adaptive merge and split to quickly locate the best solutions for large-scale problems with computationally expensive FEs, where experimental results have demonstrated the good search efficiency, scalability, and speedup of the proposed algorithm. Wang et al.[130] put forward a distributed PSO with a master-slave multigroup distributed model for coevolution, so as to improve the search efficiency and reduce the total expensive time cost. The proposed algorithm is applied to a large-scale cloud workflow scheduling application and obtains better results than compared algorithms. Furthermore, Wang et al.[131] used a master-slave multi-subpopulation distributed model for PSO to avoid local optima and accelerate optimization convergence, which is proven effective in experiments with 35 large-scale optimization problems with computational expensive FEs. Moreover, distributed algorithms can integrate with problem decomposition and resource allocation methods to cope with the large-scale challenges of computational EOP efficiently. That is, EC

algorithms can decompose the problem into several smaller-scale sub-problems and optimize sub-problems concurrently via coevolution, so as to obtain a better solution more efficiently[132]. In decomposition-based methods, it is suggested that more computational resources should be allocated to optimize sub-problems that have larger contributions to the overall problem optimization, so as to fully utilize the available computational resources wisely and efficiently. In general, methods for estimating the contributions of different sub-components can be based on moving average[132], weighted average[133], most recent contribution[134], average of recent contributions[135], predefined value function[136], sensitivity analysis[137], delta disturbance[138], Gini index[139], and multi-objective evaluation considering both fitness and diversity[140].

### 5.3 Comparisons and discussions

This part compares and discusses different parallel and distributed computation methods for EOP in recent works. According to the category in parallel and distributed computation methods for EOP, Table 4 introduces some works in three recent years, i.e., those published in IEEE Transactions from 2019 to 2021. From Table 4, we can have two observations. First, the majority of works in Table 4 are for real-world applications. This may be due to the fact that the expensive computational cost in real-world problems can help better show the benefits from parallel and distributed methods, while the benchmark problems (unless with time delay settings or large-scale characteristics) may not be computationally expensive enough to demonstrate the acceleration brought by parallel and distributed methods. Second, as shown in Table 4, parallel and distributed methods are usually used to improve search efficiency for accelerating the optimization rather than for accelerating the approximation. This suggests that the parallel and distributed methods for approximation may be insufficient in the current literature.

## 6 Real-world applications

### 6.1 Real-world case studies

As numerous EOPs arise from different real-world applications and have various challenges, many pieces of researches have been undertaken to deal with the corresponding challenges by considering the specific problem types and features, leading to application-driven methods and approaches. For example, Wang et al.[141] focused on the health care area and suggested a novel evolutionary method for the trauma system design optimization, where a large number of historical records make the FE very computationally expensive. The authors recognized that the problem is an offline data-driven problem and proposed a clustering method with multi-fidelity surrogates to achieve a balance between computational time cost and optimization accuracy. The reported results reveal that the proposed algorithm can save up to 90% of computational cost while still producing a satisfactory outcome. Differently, Guo et al.[142] researched the fused magnesium furnaces optimization, which is a typical small data-driven EOP. In this problem, the FE is expensive to conduct and only a small quantity of evaluated data may be used to develop surrogates to approximate the FE. In order to handle this challenge, a low order polynomial model is built for generating more data to help better approximate the FE. Moreover, Wang et al.[48]

Table 4 Comparisons of some recent works with different parallel and distributed computation methods

| Category | Recent works | Problem features | Parallel and distributed methods | Time complexity and speedup analysis | Year | Publication |
|---|---|---|---|---|---|---|
| Acceleration for approximation | PSAED[120] | Expensive optimization in electromagnetic design | Parallel simulations to obtain data to build approximation model | Up to 7.36 times speedup over compared methods | 2019 | *IEEE Transactions on Emerging Topics in Computational Intelligence* |
| Acceleration for optimization | RADDE[127] | Expensive optimization of the neural-network-based controller in power electronic circuit | A distributed DE with a resource-aware strategy to dispatch individuals to resources | About 2 to 4 times speedup under situations with various load burdens and states | 2021 | *IEEE Transactions on Neural Networks and Learning Systems* |
| | P³SO[128] | High-dimensional, single-objective, fitness evaluations with different time delays | A pipeline-based parallel PSO in the generational-level parallelism | Theoretical speedup can reach $\lceil NP/3 \rceil$ with $NP$ as the number of particle individuals, which has been verified experimentally | 2020 | *IEEE Transactions on Cybernetics* |
| | DGLDPSO[130] | Large-scale cloud workflow scheduling | A distributed PSO based on dynamic group learning | Time complexity is $O(MaxFEs \times D)$, where $MaxFEs$ is the total number of FEs, and $D$ is the problem dimension | 2020 | *IEEE Transactions on Cybernetics* |
| | AGLDPSO[131] | Large-scale, complexity | A distributed PSO based on adaptive group learning | Time complexity is $O(MaxFEs \times D)$ | 2021 | *IEEE Transactions on Cybernetics* |

used an active learning and surrogate-assisted algorithm to solve a transonic airfoil design optimization challenge, where evaluating potential airfoil requires computationally expensive CFD simulations. The proposed algorithm can generate a high-quality solution quickly using the active learning-based surrogate. As for the crowdshipping scheduling application problem with multiple available computationally expensive evaluation methods, the SAFE[74] method is proposed with a scale-adaptive evaluation approach to solve the problem and obtain promising results. Moreover, Chugh et al.[143] proposed a kriging-based EA to tackle main challenges in the expensive shape optimization of an air intake ventilation system, i.e., the challenges of formulating the optimization problem, connecting different simulation tools, dealing with computationally expensive objective functions, and obtaining promising results. In addition, in the blast furnace optimization problem, Chugh et al.[144] implemented principal component analysis to reduce the problem complexity and optimization difficulties, which has shown to be effective. Nevertheless, the robust optimization of an airfoil design application with various possible worst-case scenarios was treated as a multi-task optimization problem by Wang et al.[145], who proposed a surrogate model in the joint space of the decision and scenario spaces to replace some of the expensive FEs to solve the problem efficiently.

#### 6.2 Competitions and benchmarks from real-world

As mentioned above, different EOPs may favor different algorithm characteristics and features, which could not be ideal for testing and comparing new methods proposed by researchers. Therefore, some works have been conducted to provide EOP competitions and benchmarks that reveal the difficulties and challenges from real-world applications and evaluate various state-of-the-art methods, so as to bridge the gap between academic and application. For example, Table 5 lists the competitions based on real-world EOPs in the past three years. Due to the inconvenient and expensive FEs, the problems in the competitions listed in Table 5 are treated as data-driven optimization problems, where only some evaluated solutions will be provided for participants to build approximation models for optimization. Moreover, Wu et al.[74] developed 35 test instances (i.e., 25 medium-scale instances and 10 large-scale instances) for the expensive crowd shipping optimization application based on existing open-source real-world vehicle routing application instances. He et al.[146] selected seven multi-objective optimization benchmark problems from real-world applications with expensive FE, aiming to promote the research on data-driven evolutionary multi-objective optimization by suggesting a set of problems extracted from various real-world optimization applications. In addition, as design optimization problem often has an expensive and highly nonlinear objective and constraints functions, Picard and Schiffmann[147] presented a framework to design electro-mechanical actuators, called the multi-objective design of actuators, and generated 20 constrained multi-objective test problems from the framework with a specific focus on constraints for analyses and studies.

### 7 Potential research directions and open problems

Many researches have successfully implemented the idea of using EC to solve EOPs efficiently. However, despite the fact that much work has been done and the studies have been reviewed in the previous sections, we discovered that research in this field is still not systematic and that many issues remain unsolved. Therefore, this section highlights several possible research directions and open problems in the hope of inspiring further extensive and in-depth research on this topic. Specifically, as shown in Fig. 5, five potential future directions from three levels (i.e., theory-method-application level) are considered and discussed in the following contents.

#### 7.1 Deeper theoretical analysis

As EC for EOP has become more complex by incorporating the problem approximation, novel operators, and

Table 5 Competition problems from expensive real-world applications in three recent years

| Competitions | Description | Year | Links |
|---|---|---|---|
| Competition at IEEE congress on evolutionary computation | Seven benchmark multi-objective optimization problems from real-world applications, where the evaluations are expensive | 2019 | https://mp.weixin.qq.com/s/0QjFs3zl-oZ_0i-Ww3hbIQ// or https://github.com/HandingWang/DDMOP |
| Competition at IEEE world congress on computational intelligence | Eight benchmark problems from two real-world applications of aerodynamic optimization and software configuration tuning | 2020 | https://mp.weixin.qq.com/s/02FTnctkC6l-ssUNSAqKJA// or https://github.com/HandingWang/DDEO-WCCI2020 |
| Competition at IEEE congress on evolutionary computation | Six problems of parameter optimizations of flocking model for a swarm of robots in different scenarios | 2021 | https://handingwang.github.io/DDEO-CEC2021/ |

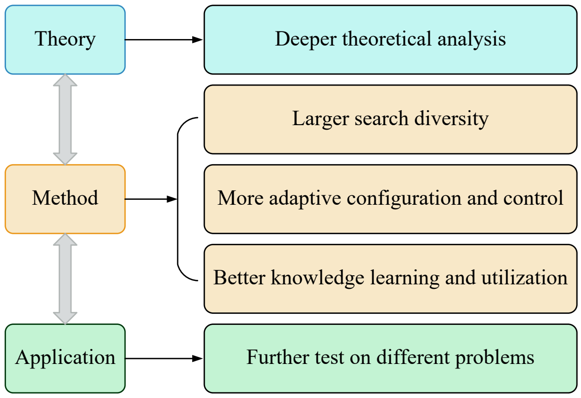

Fig. 5 Potential future research directions and open issues parallelization methods, it will be more difficult to clarify whether the trade-off between optimization accuracy and optimization efficiency will be acceptable and how the trade-off is achieved. In addition to the numerical studies based on benchmark functions, it may be essential to make deeper theoretical analyses on the new EC algorithms for EOP, so as to clarify the influences of incorporating methods in the search procedure of EC. Therefore, it could be a worthwhile research direction to conduct a deeper theoretical analysis on the components of the proposed EC.

### 7.2 Larger search diversity

As approximation methods may have approximation bias and uncertainty in some search areas, populations with smaller diversity may be trapped in local optima indicated by the approximation models. Therefore, it could be a potential research direction to improve the search diversity of algorithm to reduce the negative influence of the approximation model as much as possible.

### 7.3 More adaptive configuration and control

Due to the expensive FEs, it would be impossible to configure the parameters of both EC and approximation models for a new problem via a trial-and-error manner. Therefore, the adaptive and automatic configuration and control of the algorithm including the surrogate model management would be a necessary research direction for obtaining algorithms that can work well for various emerging problems and applications.

### 7.4 Better knowledge learning and utilization

The expensive FE cost poses great challenges in obtaining useful knowledge and information from the problems for improving the search process. Therefore, it would be appealing to consider advanced knowledge learning and utilization approaches to improve EC, such as few-shot learning, self-supervised learning, and knowledge transfer from related problems and scenarios.

### 7.5 Further test on different problems

The good performance of EC for EOP is mainly studied on continuous problems and is mainly in the field of several kinds of optimization problems, such as the multi-objective and constrained problem. However, the application of EC in more complex problems is still insufficient, such as problems with discrete and combinational[148–150], multitask[151], dynamic[152], many-objective[153, 154], and multi-modal characteristic[155–157]. Moreover, powerful EC is also needed for complex problems with hybrid difficulties, such as combinational multi-objective difficulty[158, 159], constrained multi-objective difficulty[160–162], and sparse multi-objective difficulty[163, 164]. Therefore, exploring how to design effective and efficient EC algorithms for these complex problems could be a potential research direction in the near future.

## 8 Conclusions

This article has provided a comprehensive survey on EC for EOP. In this paper, analysis has been provided mathematically on the total optimization cost of using EC to solve EOPs, based on which three research directions have been pointed out for reducing total cost. The taxonomy of researches has been defined by considering the three directions and the application. As a result, the taxonomy contains four parts: problem approximation and substitution, algorithm design and enhancement, parallel and distributed computation, and real-world applications. In each part, existing related works are further classified and introduced. Therefore, such a systematic taxonomy can offer a better understanding of why and how EC algorithms have been used to solve EOPs efficiently and provide references to help researchers in various fields to solve EOPs more efficiently. Based on the review of the existing works, some potential directions and open problems have also been proposed and discussed for this area, so as to provide inspiration to the ongoing development of the related research fields. We hope that this survey can help systematize existing work, evoke increased interest, inspire new ideas, and expand the scope of research in this new, exciting, and fascinating research area.

## Acknowledgments

This work was supported by National Key Research and Development Program of China (No. 2019YFB2102102), the Outstanding Youth Science Foundation (No. 61822602), National Natural Science Foundations of China (Nos. 62176094, 61772207 and 61873097), the Key-Area Research and Development of Guangdong Province (No. 2020B010166002), Guangdong

Natural Science Foundation Research Team (No. 2018B030312003), and National Research Foundation of Korea (No. NRF-2021H1D3A2A01082705).

### Open Access

This article is licensed under a Creative Commons Attribution 4.0 International License, which permits use, sharing, adaptation, distribution and reproduction in any medium or format, as long as you give appropriate credit to the original author(s) and the source, provide a link to the Creative Commons licence, and indicate if changes were made.

The images or other third party material in this article are included in the article's Creative Commons licence, unless indicated otherwise in a credit line to the material. If material is not included in the article's Creative Commons licence and your intended use is not permitted by statutory regulation or exceeds the permitted use, you will need to obtain permission directly from the copyright holder.

To view a copy of this licence, visit http://creativecommons.org/licenses/by/4.0/.
